# Adoption of ‘decentralized’ clinical trial terminology: a systematic review of self-identified decentralized clinical trials

**DOI:** 10.1186/s13063-026-09922-x

**Published:** 2026-07-31

**Authors:** Annie Wright, Otavio Berwanger, Shwetha Rajaram, Joanna Gambell, Abdul Salam, Victoria Cornelius

**Affiliations:** 1 Imperial College London, London, UK; 2 George Institute for Global Health UK, London, UK; 3 George Institute for Global Health India, New Delhi, India; 4 University of New South Wales, Sydney, Australia

**Keywords:** Decentralized clinical trials, Decentralized elements, Decentralization

## Abstract

**Importance:**

In recent years, trials have leveraged digital tools and remote methods to improve patient access and diversity, streamline operations, enhance data collection, and reduce costs and timelines. Regulatory terminology has defined these as *decentralized clinical trials*. While many earlier trials incorporated one or more decentralized elements (e.g. e-consent, remote monitoring), little is known about recent trials that adopt multiple elements and explicitly identify as decentralized.

**Objective:**

To determine, in trials explicitly identifying as decentralized, which decentralized elements they use, where they are occurring, and which therapeutic areas and interventions they involve.

**Methods:**

A search of MEDLINE, Embase, and CENTRAL from inception to September 9, 2024, yielded 4357 articles. Two reviewers independently and in duplicate screened titles, abstracts, and full texts to identify trials explicitly described as decentralized. Key data were extracted in duplicate to ensure accuracy. Additionally, the International Clinical Trials Registry Platform and ClinicalTrials.gov were searched on July 18, 2024, to identify unpublished and ongoing decentralized clinical trials.

**Results:**

Thirty-three publications (28 unique trials) met inclusion, all published since 2021, with numbers increasing annually. Only four trials described themselves as decentralized in their trial registration records. Of the 28 included trials, 54% were completed and 46% were ongoing, with a median sample size of 900 (IQR 192-1470). All were single-country studies, 96% from high-income countries, mostly in North America. Pharmacological interventions were most common (43%). Among nine decentralized components evaluated, e-consent was used in 71% of trials, and devices for outcome data collection in 75%. It was unclear if electronic health records were used for screening in 75%, and centralized monitoring in 79% of trials. Patient-reported outcomes were frequent, collected in 86% of trials and serving as the primary outcome in 61%.

**Conclusion:**

Since regulators introduced the terminology in 2018, adoption among trials explicitly identifying as decentralized has increased since 2021, although the overall number remains low. These trials occur mainly in high-income countries, suggesting potential inequities in implementation or reporting. No single decentralized element combination dominated, indicating variability in how decentralized approaches are implemented across disease areas and interventions. Improved reporting is needed to distinguish between underuse and underreporting of decentralized elements.

**PROSPERO registration number:**

CRD42024571898.

**Supplementary Information:**

The online version contains supplementary material available at https://doi.org/10.1186/s13063-026-09922-x.

## Introduction

Decentralized Clinical Trials (DCTs) are defined as studies where one or more aspects of their conduct occur outside a traditional setting, such an academic site [1]. The use of DCTs has been on the rise, and the COVID-19 pandemic transformed the way trials are conducted, making decentralization a necessity. Conducting DCTs has the potential to provide several benefits both for those participating in the trials and those overseeing them. In this sense, the use of DCTs can potentially reduce the carbon footprint of trials, improve participant recruitment and retention due to their patient-centricity, increase patient diversity, and reduce timelines and trial-related costs [2–4].

Decentralized elements refer to trial processes that have been decentralized, e.g. using e-consent rather than consenting in person or blood sample collection taking place in the participant’s home rather than at a trial site. Decentralized data collection methods, such as the use of routine data sources, wearable devices, and electronic patient-reported outcome measures (ePROMs), enable easier collection of longitudinal data to improve trial efficiency. Operationally, traditional clinical trials are inefficient and DCTs can offer alternatives to how these processes are typically carried out [5]. While many trials may use single decentralized elements, those fully exploiting decentralization offer the potential to connect patient-centred and resource-efficient trials.

The terminology surrounding remote clinical trials has evolved significantly over the past 7 years. Santa Ana Tellez et al. highlighted the proliferation of inconsistent terminology including ‘virtual’, ‘remote’, ‘digital’, and ‘hybrid’ trials, emphasizing the urgent need for harmonization in professional communication [6]. Since 2018, ‘decentralized clinical trial’ has been increasingly used in research articles and regulatory guidance documents, with derivatives such as ‘remote decentralized clinical trial’ introduced by the Trials@Home consortium in 2019 [6]. Subsequently, regulatory agencies have increasingly adopted ‘decentralized’ as the preferred standardized term: the EMA issued DCT guidance in 2022, FDA published comprehensive DCT guidance in 2024, while the International Council for Harmonisation (ICH) incorporated decentralized elements into ICH E6(R3) guidelines [1, 7, 8]. This regulatory convergence around ‘decentralized’ terminology represents a critical inflection point in professional consensus, making systematic examination of self-identified DCTs methodologically important for understanding field standardization and regulatory alignment.

While previous systematic reviews have comprehensively examined remote trial methods using a broad range of related terms (e.g. remote, online, digital), no review has specifically examined trials that explicitly self-identify as decentralized [9]. We specifically included only trials explicitly described as ‘decentralized’ to capture self-identification patterns rather than researcher-imposed categorization. Given the regulatory emphasis on ‘decentralized’ terminology following several guidance papers, this approach captures alignment with current regulatory frameworks rather than historical remote trial approaches.

In this review, we aimed to systematically examine trials that are designed to be decentralized and determine which elements they use, where these trials are occurring, and what therapeutic areas and types of interventions they involve.

## Methods

Preferred Reporting Items for Systematic Reviews and Meta-Analyses (PRISMA) guidelines were adhered to for the reporting of this systematic review [10]. The protocol was prospectively registered on PROSPERO (CRD42024571898) [11].

### Eligibility criteria

The inclusion criteria were randomised controlled trials (RCTs) either ongoing (the protocol is published) or completed (published results) and explicitly self-identified as decentralized, conducted in any population, evaluated any intervention, and had a clinical outcome. Trials were excluded if they incorporated some decentralized elements, such as e-consent, but were not set out (not explicitly mentioned) to be decentralized as this review aims to evaluate terminology adoption and regulatory alignment. Only trials published in English were included.

### Search strategy

A systematic search to identify trials (protocols or results) published before September 9, 2024 (inclusive), was performed in MEDLINE, Embase, and CENTRAL. Search terms were truncation of ‘decentralized’ and MeSH term terms and synonyms of RCT (eAppendix1–3). Other terms, such as ‘virtual’ trials, were not included as they do not fully encompass the range of decentralized elements that are not digitally focused [9].

To find unpublished and ongoing trials, the International Clinical Trials Registry Platform and Clinicaltrials.gov were searched on July 18, 2024, and any records using the term ‘decentralized’ (truncated) were extracted. This search was included to identify self-identified DCTs that had not yet been published and to provide additional insight into ongoing and recently initiated DCT activity.

### Data selection and extraction

Articles were imported into COVIDENCE; duplicates were removed, for screening, title abstract, and full-text. COVIDENCE auto-remove non-RCTs feature was used and checked by the first reviewer to ensure no studies were incorrectly excluded. Two reviewers screened, title and abstracts and full texts, and reviewers met to resolve disagreements by consensus.

eTable 1 details the variables extracted. Extraction was completed in duplicate by two reviewers independently. For platform trials with several publications, the earliest publication was used for data extraction. Where both a protocol and results were available for the same trial, the results paper was used as the primary source for data extraction and supplemented by the protocol. Where only results publications were identified, systematic searches for trial protocols and/or statistical analysis plans were performed to minimise missing data. All materials utilised for each trial are detailed in the supplemental material (eTable 2).

Trials identified from the registry search were assessed for eligibility by the first reviewer.

### Data items

Data were extracted on study design, location, therapeutic areas, and use of prespecified decentralized elements (Yes/No/Not Reported/Unclear/Not Collected). Data were extracted on nine prespecified decentralized elements: use of electronic health records (EHR) for screening, e-consent, physiological sample collection, biological sample collection, centralised monitoring, use of EHR for outcome collection, use of a device for outcome collection, investigational medicinal product (IMP) delivery, and use of an online adverse event reporting platform (Fig. 3). We identified the type of outcome collection method employed (e.g. patient-reported outcome measures (PROMs), wearable devices, or routinely collected data).

For those trials identified from the clinical trials registries, the type of intervention and location of the study were extracted. The reason they did not appear in the database search was extrapolated, i.e. there are no associated publications, or the trial is ongoing (recently started). As registry records do not provide the same level of methodological and reporting detail as published protocols and results papers, these data were summarised separately from the main review findings.

### Risk of bias assessment

A modified risk of bias assessment was conducted using the Cochrane Risk of Bias (RoB2) tool for randomised trials to assess the quality of key design and conduct features. A full RoB was not performed as this is a methodology review where comparison of the outcomes was not the primary focus [12]. The risk of bias was evaluated based on pre-specified domains: randomisation process, deviation from the intended interventions, and measurement of the outcome. Other RoB2 domains, missing outcome data and selection of the reported result, were not assessed as they were outside the scope of this review. Therefore, these assessments should not be interpreted as a full appraisal of internal validity or overall risk of bias of the included trials.

### Statistical analysis

Evidence from the included trials was presented through both descriptive and narrative summaries. Trial characteristics were tabulated with frequencies and percentages or medians and interquartile ranges where applicable. A heatmap was created to visualize the common combinations of decentralized elements in trials and to assess trends over time. There was no handling of missing data as only summaries are presented. All analyses were performed using R version 4.3.1.

## Results

**Fig. 1:**
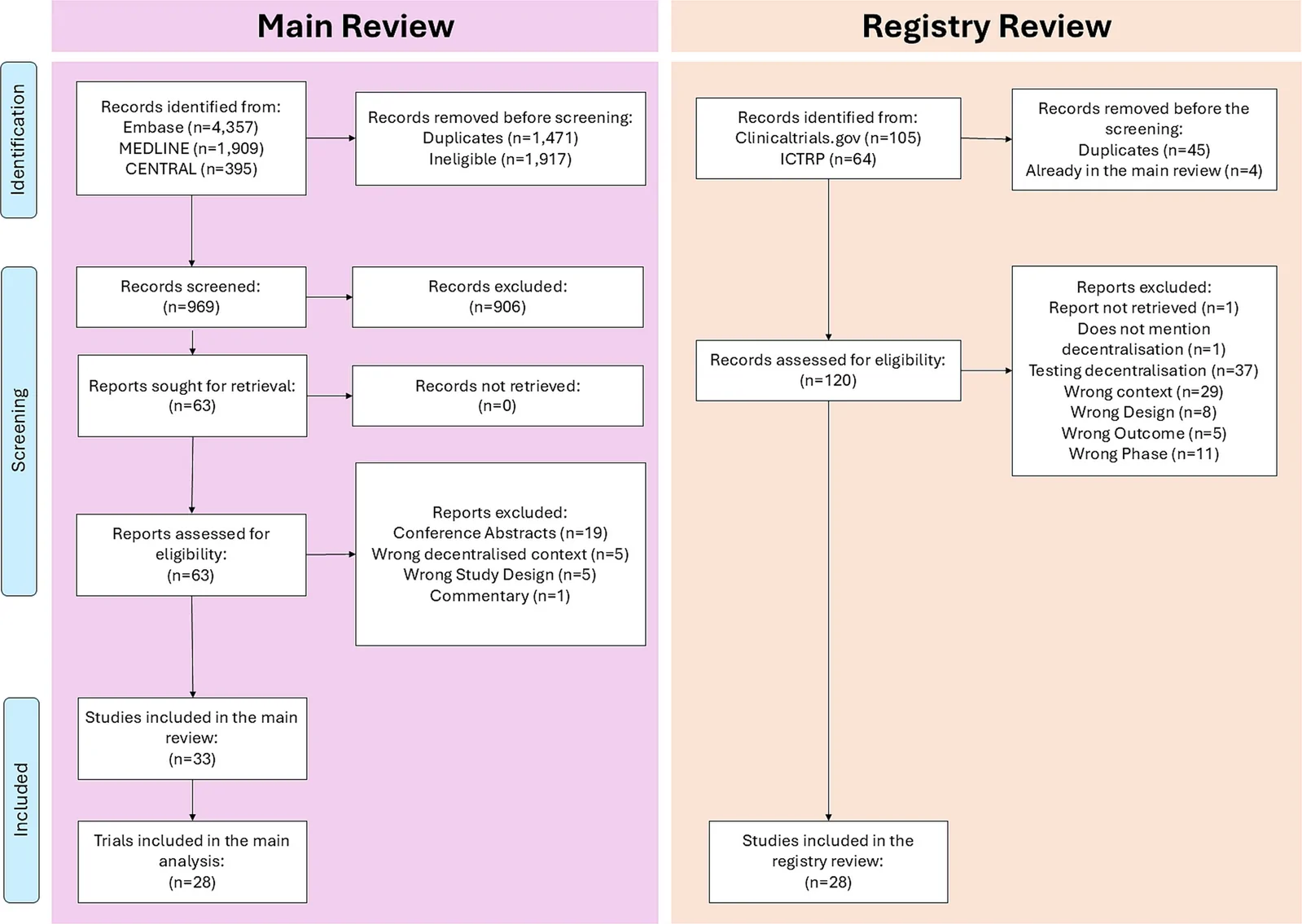
Study selection process

A total of 4,357 records were identified, of which 33 (0.76%) were included [13–40], PRISMA Flow (Fig. 1). Of the included records, 28 unique trials were identified, 13 were ongoing (protocol published), and 15 were completed (results published). Details on the papers that were extracted are in Table 1.

**Table 1:** Summary characteristics of included trials

Study ID	DCT classification	Therapeutic area	Intervention/control	Target population (years)	Location	Expected sample size	Number randomised (% of expected sample size)
Trials in progress							
Akechi 2024 [13]	Fully remote	Mental health	8-week smartphone-based intervention vs. waitlist control	Children/adults (15–39)	Japan	224	Not applicable as recruitment ongoing
Best 2023 [16]	Combination of central clinic and home	Neurology	Prenatal multivitamin and mineral supplement vs. standard of care	Pregnant women (> 16)	Australia	754	
Gibson 2023 [20]	Fully remote	Cardiovascular	Core digital engagement program (CDEP) via iPhone application and an Apple vs. CDEP alone	Adults (≥ 65)	United States	28,000	
Huckvale 2023 [21]	Fully remote	Mental health	Self-directed application vs. active control	Adults (≥ 18)	Australia	1440	
Krumholz 2024 [25]	Fully remote	Infectious diseases	Nirmatrelvir/ritonavir vs. placebo/ritonavir	Adults (≥ 18)	United States	100	
LaForce 2024 [26]	Fully remote	Respiratory	Inhaler vs. standard of care	Children/adults (≥ 12)	United States	2500	
Naik 2024 [32]	Combination of central clinic and home	Neurology	Low-dose naltrexone vs. placebo	Adults (19–69)	Canada	160	
Schröder 2022 [33]	Fully remote	Neurology	DTx sinCephalea vs. standard of care	Adults (18–65)	Germany	512	
Shortt 2024 [34]	Combination of central clinic and home	Other	ECMT-154 vs. placebo	Adults (18–65)	New Zealand	118	
Silberman 2022 [36]	Fully remote	Respiratory	Digital asthma self-management (DASM) program vs. other	Adults (18–64)	United States	900	
Spertus 2021 [38]	Fully remote	Cardiovascular	Canagliflozin vs. placebo	Adults (≥ 18)	United States	1900	
Yao 2024 [39]	Fully remote	Cardiovascular	Apple watch vs. standard of care	Adults (≥ 18)	United States	1420	
Yokoi 2024 [40]	Combination of central clinic and home	Neurology	Photobiomodulation vs. sham	Adults (60–90)	Japan	30	
Completed							
Akechi 2024 [14]	Fully remote	Mental health	Smartphone-based intervention vs. other	Adults (≥ 20)	Japan	352	359 (102)
Akechi 2022 [15]	Fully remote	Mental health	Smartphone-based problem-solving therapy vs. waitlist	Adults (20–49)	Japan	444	447 (101)
Boucher 2024 [17]	Fully remote	Mental health	Happify application vs. waitlist	Children (13–17)	United States	800	353 (44)
Bramante 2023 [18]	Fully remote	Infectious diseases	Metformin, ivermectin, fluvoxamine vs. placebo	Adults (30–85)	United States	1350	1431 (106)
Chung 2024 [19]	Unclear	Endocrine	Application, nutritional intervention, application, and nutritional intervention vs. unclear	Adults (≥ 18)	South Korea	Not reported	175 (NA)
Kaizer 2023 [22]	Fully remote	Infectious diseases	Lopinavir/ritonavir vs. placebo	Adults (≥ 18)	United States	900	448 (50)
Kolobaric 2024 [23]	Fully remote	Other	TruCBN vs. placebo	Adults (≥ 21)	United States	1500	1020 (68)
Kolobaric 2023 [24]	Fully remote	Other	THC and botanicals vs. placebo	Adults (≥ 21)	United States	900	620 (69)
Lee 2021 [27]	Fully remote	Respiratory	Messaging (educational and call to action) vs. other	Adults (≥ 18)	United States	8086	31,404 (388)
Lu 2023 [28]	Fully remote	Mental health	Acceptance and commitment therapy vs. waitlist	Adults (≥ 18)	China	137	145 (106)
Mackenzie 2022 [29]	Fully remote	Cardiovascular	Dosing regime vs. other	Adults (≥ 18)	United Kingdom	20,000	21,104 (106)
Marshall 2022 [30]	Fully remote	Respiratory	Messaging vs. other	Adults (≥ 18)	United States	49,000	49,138 (100)
Naggie 2022 [31]	Combination of central clinic and home	Infectious diseases	Ivermectin (400 μg/kg) vs. placebo	Adults (≥ 30)	United States	1200	1800 (150)
Shortt 2022 [35]	Combination of central clinic and home	Other	Kanuka oil vs. placebo	Adults (18–65)	New Zealand	80	80 (100)
Silverberg 2024 [37]	Combination of central clinic and home	Other	Crisaborole ointment (2%) vs. other	Adults (≥ 45)	United States	66	66 (100)

### Trial characteristics

**Fig. 2:**
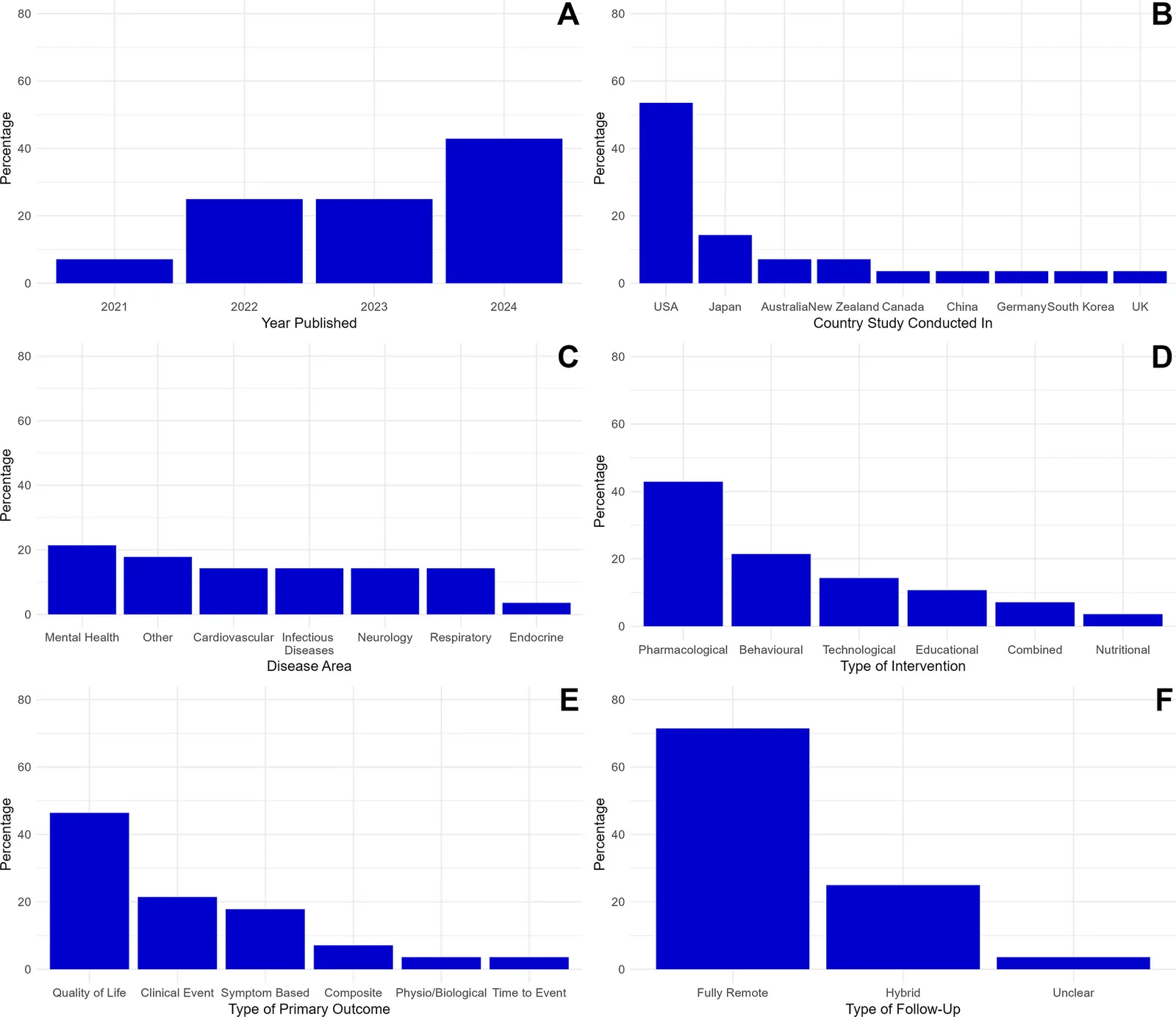
**A**–**F** Summary of the key information of the trials included in the main review (*n* = 28). **A** Year of publication. **B** Country where the study was conducted. **C** Disease area. **D** Type of intervention. **E** Type of primary outcome. **F** Type of follow-up

Figure 2 and eTable 3 outline the characteristics of the included trials. Trials were published between 2021 and 2024 with the number increasing over time. These trials were first registered on a clinical trials registry between 2018 and 2024 with one outlier in 2014. Of the 28 trials included, 54% (*n* = 15) were results publications, while 46% were protocols of ongoing trials.

Most trials (54%) were ongoing or completed in the USA and only one was not conducted in a high-income country. All trials were conducted within a single country. Mental Health was the most common therapeutic area (21%). Four trials were conducted in each cardiovascular, infectious diseases, neurology, and respiratory, respectively (Fig. 2, eTable 3).

Twelve trials (43%) evaluated a pharmacological intervention, six (21%) a behavioural intervention, and four (14%) a technological intervention (e.g. a smartwatch) with the remaining six being combination, nutritional, or educational (Fig. 2, eTable 3). Quality-of-life outcomes were used as the primary endpoint in 46% of the trials, but the specific types of quality-of-life measures varied.

A parallel-arm design was used most frequently (*N* = 25, 89%), and three were platform trials. Notably, 71% (*N* = 21) of the trials were conducted entirely remotely, with no in-person visits. Other trials used a hybrid approach with some visits in-person (25%, *N* = 7).

The median trial enrolment period of completed trials was 11.1 months (IQR 0.7–77.6). The median planned sample size for these trials was 900 (IQR 30–49,000), with three large trials with sample sizes of 20,000, 28,000, and 49,000 [20, 29, 30]. The median number of participants randomised was less at 448 (IQR 66–49,100) with four trials (27%) under-recruiting compared to their calculated sample sizes and eight trials (53%) overrecruiting. The median over-recruitment was 106% (102%–117%), and the median underrecruiting was 59% (48%–68%).

### Use of decentralized elements

The reporting of decentralized elements was inconsistent, with various terms used for each type, making it unclear whether they were not used or simply not reported. In ongoing trials, more elements were reported.

**Fig. 3:**
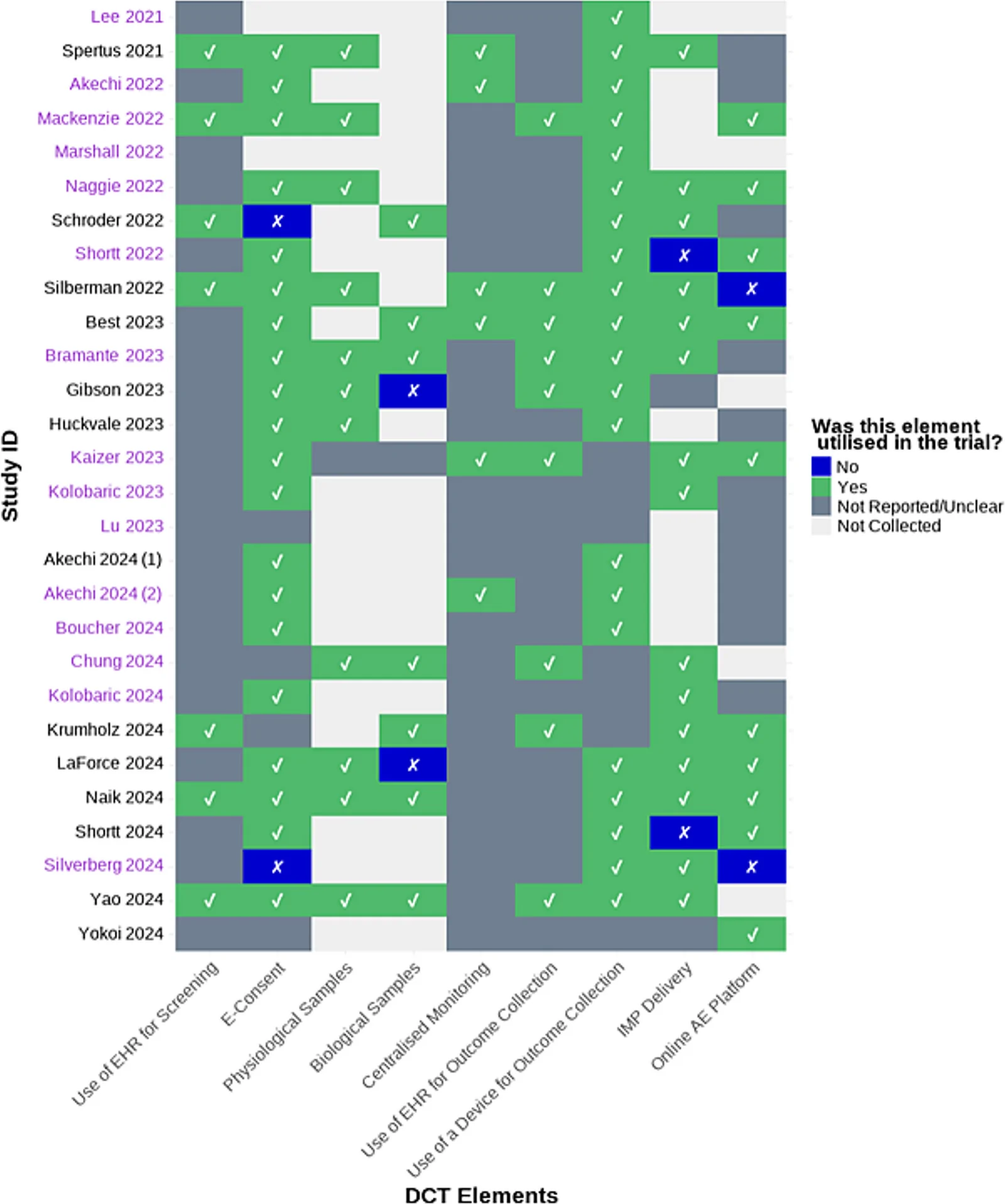
Commonly used elements in DCTs. Black study IDs are ongoing trials and purple are completed trials. ‘Not Collected’ refers to cases where the action related to an element was carried out, e.g. there were no biological samples collected in the trial at all, and thus, this element could not be used in the trial. ‘Not Reported/Unclear’ refers to when the element was not explicitly mentioned in the paper, but it could not be discounted that this element was not used

Except for the study by Lu et al., all trials utilised at least one of the pre-specified decentralized elements, and five trials incorporated seven of these elements (Fig. 3, eTable 4). Overall, no clear trends were observed over time regarding the use of specific decentralized elements or the number of elements incorporated in a trial.

Electronic health records were used for screening in seven trials (25%) and outcome collection in nine trials (32%). E-consent was deployed in 20/26 trials (77%) that used consent; two trials had obtained a waiver of informed consent.

In trials where physiological or biological samples were collected (*n* = 12 and *n* = 10, respectively), 92% of physiological samples and 70% of biological samples were collected in the participant’s home.

The use of centralised monitoring was only explicitly mentioned in six trials (21%).

The use of a device for outcome collection was the most common element across all trials, with 21/28 trials (75%) utilizing it. These devices ranged from mobile apps for patient-reported outcomes to pedometers for activity tracking. In trials involving an IMP or device (*n* = 19), 15 (79%) delivered it directly to participants’ homes, while two trials (11%) required participants to collect it in person.

Online adverse event platforms for participants to directly report adverse events to the trial team between visits were available in 10/23 trials that collected adverse events (48%).

### Data collection methods

Table 2 outlines the data collection methods used in the trials. For the primary outcome measure, patient-reported outcome measures (PROMs) were the most common method (61%). PROMs were also used for secondary outcomes in 85% of the included trials.

**Table 2:** Data collection methods in included trials

	Clinical event (*N* = 6)	Composite (*N* = 2)	Physio/biological (*N* = 1)	Quality of life (*N* = 13)	Symptom based (*N* = 5)	Time to event (*N* = 1)	Overall (*N* = 28)
Primary outcome collection method							
Case report forms	4 (66.7%)	0 (0%)	0 (0%)	0 (0%)	1 (20.0%)	0 (0%)	5 (17.9%)
Patient reported outcome measures	0 (0%)	1 (50.0%)	0 (0%)	13 (100%)	3 (60.0%)	0 (0%)	17 (60.7%)
Routinely collected data and case report forms	1 (16.7%)	0 (0%)	0 (0%)	0 (0%)	0 (0%)	0 (0%)	1 (3.6%)
Routinely collected data	1 (16.7%)	1 (50.0%)	1 (100%)	0 (0%)	0 (0%)	1 (100%)	4 (14.3%)
Unclear	0 (0%)	0 (0%)	0 (0%)	0 (0%)	1 (20.0%)	0 (0%)	1 (3.6%)
Were patient reported outcome measures collected at all?							
No	3 (50.0%)	1 (50.0%)	0 (0%)	0 (0%)	0 (0%)	0 (0%)	4 (14.3%)
Yes	3 (50.0%)	1 (50.0%)	1 (100%)	13 (100%)	5 (100%)	1 (100%)	24 (85.7%)
What was the method of patient reported outcome measures collection?							
Electronic	3 (100%)	1 (100%)	1 (100%)	12 (92.3%)	5 (100%)	1 (100%)	23 (95.8%)
With psychologists	0 (0%)	0 (0%)	0 (0%)	1 (7.7%)	0 (0%)	0 (0%)	1 (4.2%)
Was routinely collected data used at all?							
Not reported	4 (66.7%)	0 (0%)	0 (0%)	11 (84.6%)	4 (80.0%)	0 (0%)	19 (67.9%)
Yes	2 (33.3%)	2 (100%)	1 (100%)	2 (15.4%)	1 (20.0%)	1 (100%)	9 (32.1%)
Was a device used at all?							
Not reported	0 (0%)	0 (0%)	1 (100%)	4 (30.8%)	1 (20.0%)	0 (0%)	6 (21.4%)
Unclear	0 (0%)	0 (0%)	0 (0%)	1 (7.7%)	0 (0%)	0 (0%)	1 (3.6%)
Yes	6 (100%)	2 (100%)	0 (0%)	8 (61.5%)	4 (80.0%)	1 (100%)	21 (75.0%)
Was the device used to aid the primary outcome collection?							
No	5 (83.3%)	2 (100%)	NA	8 (100%)	4 (100%)	1 (100%)	20 (95.2%)
Yes	1 (16.7%)	0 (0%)	NA	0 (0%)	0 (0%)	0 (0%)	1 (4.8%)

Routinely collected data was used in four trials specifically for the primary outcome (14%) and for a further five trials for other secondary or exploratory outcomes (32%).

A device was used in 75% of trials to aid the collection of outcome data; however, the device was only actually used for the primary outcome in one trial (5%). An app on a mobile device was the most common (*n* = 10) followed by a wrist-worn device (*n* = 3).

### Clinical trial registries

One hundred twenty-four trials were identified from clinical trial registries (Fig. 1). Of these, 92 were not considered to be eligible as per the inclusion and 28 were deemed eligible and could have been included had they been found in the database search. Of the ineligible trials, 40% were ineligible as they were testing the decentralization of a procedure, and 32% were using the term ‘decentralized’ in the wrong context such as ‘decentralizing antiretroviral therapy care delivery’ (eTable 5).

Of the 28 trials identified from the registry search, 15 were ongoing (54%) (eTable 6). Just over a quarter of the trials (29%) had been completed but did not have any associated publications available.

The type of interventions investigated in these registry trials showed a different distribution compared to those included in the review (eTable 6). Twenty-five percent of interventions were pharmacological, 25% were behavioural interventions, 21% were combined interventions, and 11% were technological interventions. The geographical distribution of studies was 25% conducted in Asia, 46% in North America, and 18% in Europe.

Only four of the 28 trials included in the main review were found in the search of the registry databases indicating that the 24 trials did not use decentralized to describe themselves upon database registration.

### Risk of bias evaluation

The risk of bias assessment was conducted for all 28 included trials (eTable 7). Most trials (46%) were judged to have a low risk and 32% were viewed to have some concern of bias overall, mostly due to participants or carers or people delivering the interventions being aware of the assigned intervention during the trial. Twenty-one percent of trials were deemed to have a high risk of bias overall, primarily due to the lack of blinding.

## Discussion

### Main findings

This systematic review of 28 published DCTs provides an overview of trials that are designed to be decentralized, determining which elements they use, where these trials are occurring, and what therapeutic areas and types of interventions they involve. Our findings show that trials explicitly identifying as ‘decentralized’ emerged exclusively from 2021 onwards, 3 years after the term gained prominence in research literature and regulatory documents in 2018. This temporal pattern reflects broader regulatory harmonization efforts and suggests a maturation period where terminology evolved from conceptual frameworks into practical implementation in registered clinical trials. Our cataloguing of decentralized components reveals implementation priorities within the evolving regulatory framework. The frequent use of e-consent (71%) and use of devices for data collection (75%) suggests these elements align well with current regulatory guidance and practical feasibility. However, the underreporting of elements such as electronic health records for screening participants (75%) and centralized monitoring (79%) may indicate the lack of reporting guidance while underuse, which is less common, might be due to ongoing regulatory, technical, or practical barriers. Lack of reporting guidance is also highlighted by the fact that only four of the included studies described themselves as DCTs in their trial registration.

This review found that many of the DCTs are US-based with no current publications of multi-country studies and only one conducted outside of a high-income setting. The geographic concentration in high-income countries may partly reflect varying regulatory landscapes, greater availability of funding streams, for innovative trial models, and infrastructure requirements. The absence of multi-country DCTs suggests ongoing regulatory harmonization challenges across different jurisdictions. While FDA guidance applies primarily to US studies, the ICH framework influences global drug development [1, 8]. However, regional regulatory differences, such as the 27-country complexity addressed by EMA guidance versus single-jurisdiction FDA guidance, may contribute to implementation variations across regions [1, 7]. The geographic concentration of included studies may reflect English-language publication bias and emphasis on US/European regulatory frameworks.

### Comparison to previous research

We observe that most of the included trials are using e-consent when consent was required (77%). This may have been a necessity out of the COVID-19 pandemic or indicate the shift in the use of technology in clinical trials. Jong et al. found that decentralized consenting increased from 4.2% in early 2019 to 20.9% in early 2020 whilst on-site consenting decreased [41]. A recent survey of clinical trials units in the UK found that 62% of them had already implemented e-consent in at least one of their trials and 54% of those had not, planned to in the next 6–12 months indicating a trend to the use of e-consent [42].

Digital health technologies enable a wealth of new ways of collecting data within trials which can provide more dynamic data, in-depth insights, and strengthen evidence when done well [43]. The impact of using digital health technologies for outcome collection is beneficial to patients and intrinsically linked to the proposed benefits of decentralization as a whole so it is promising that the results show that adoption [44]. Jong et al. found that decentralized data collection was reported in 68.9% of protocols, the most utilized decentralized procedure, which is similar to our findings, where it was explicitly used in 75% of trials [41].

Previous reviews have examined DCT methods using broad inclusion criteria, including Rogers et al. who identified 45 studies using any remote trial approaches [9, 45]. In contrast, our review specifically examines trials that explicitly self-identify as ‘decentralized’, capturing regulatory terminology adoption rather than methodological categorization. This methodological difference produces some notable variations in findings. Rogers et al. found e-consent in 33% of their broadly defined remote trials, while 77% of explicitly self-identified DCTs in our review used e-consent. Geographic distribution was primarily concentrated in North America in both reviews, with our findings showing 96% of trials conducted in high-income countries more broadly.

Even among trials that self-identify as decentralised, terminology and reporting are inconsistent, making identification, interpretation, and synthesis of DCT evidence challenging. This deliberate focus on self-identified decentralised trials was chosen to examine how decentralised trials are defined and presented by investigators when explicitly labelled as such, under the assumption that these represent the most clearly articulated examples of decentralised trial design. Even within this group, we observed substantial variability in reporting completeness and terminology use, suggesting that trials not explicitly labelled as decentralised are likely to present even greater challenges for evidence synthesis. While our findings complement Rogers et al.’s methodological review, they address a different research question. Rogers et al. examined what methods are used for decentralization, while we examined how trials that explicitly identify as decentralized implement these approaches. This provides additional perspective on regulatory terminology adoption and implementation patterns in the evolving DCT landscape.

### Implications of our findings

The exclusive emergence of explicitly ‘decentralized’ clinical trials since 2021 demonstrates how regulatory guidance has successfully shaped professional terminology and research positioning. This shift from the varied terminology documented by Santa Ana Tellez et al. to standardized ‘decentralized’ language reflects meaningful professional consensus formation, with practical implications for how trials are funded, regulated, and adopted [6].

Our finding that 96% of self-identified DCTs were conducted in high-income countries suggests important equity considerations, although this may also reflect publication and language bias. This pattern is particularly notable given that non-communicable diseases account for 80% of deaths in low- and middle-income regions [46]. Certain decentralised trial elements, including remote data collection and electronic consent, may be more feasible in resource-limited settings and could support improved trial accessibility.

The variable implementation of decentralized components provides benchmark data for current adoption patterns. High use of e-consent (77%) and use of a device for outcome collection (75%) suggests regulatory alignment, while underreporting of electronic health records for screening (25%) may indicate remaining implementation barriers or poor reporting of lesser-known elements.

Our systematic examination of self-identification patterns complements existing methodological reviews by revealing how regulatory standardization influences research communication. Notably, only four of the 28 included trials described themselves as decentralised in their trial registration records despite subsequently self-identifying as decentralised in a protocol or results publication. This discrepancy highlights inconsistencies in the use of decentralised trial terminology across reporting sources and may reduce the discoverability of decentralised trials in registry-based searches and evidence syntheses. The identification of ongoing trials through registries suggests continued evolution in this space, emphasizing the need for tracking both terminology adoption and implementation practices to understand field maturation. These findings support the development of clearer reporting standards that capture both methodological approaches and professional positioning within evolving regulatory frameworks.

### Limitations

This review of the literature found relatively few trial publications explicitly describing themselves as decentralized, despite decentralization being a prominent topic. Additional trials identified as decentralized were limited to conference proceedings or abstracts, which lacked sufficient detail for data extraction. Our focus on explicit ‘decentralized’ terminology means earlier remote trials using different terminology (pre-regulatory harmonization) were excluded. It is also possible that in some cases the term ‘decentralised’ was removed during editorial or peer review processes prior to publication. However, this terminological choice reflects meaningful regulatory evolution in the naming of these trials rather than an arbitrary restriction, as documented by Santa Ana Tellez et al.’s call for terminological standardization [6]. Our systematic approach captures professional self-identification patterns and evaluates terminology adoption rather than comprehensive method usage, complementing but not replacing broader methodological reviews like Rogers et al. [9].

Several decentralised trial elements were underreported in the included studies, particularly the use of EHRs for participant screening and centralised monitoring. The reasons for this limited reporting are unclear. It is possible that these activities, and their traditional trial counterparts, are routinely undertaken but not considered sufficiently important to describe in trial publications. To gain a broader understanding of the implementation of decentralised trial elements, future research could directly survey individuals involved in the design and delivery of DCTs.

Further research could also explore the need for, and perceived value of, a standardised reporting framework for decentralised trial elements. Such guidance could potentially be incorporated into clinical trial registries or developed as an extension to existing reporting standards, such as CONSORT. However, the development and implementation of such a framework would require substantial collaboration across multiple stakeholder groups.

## Conclusion

In summary, the present systematic review provides a systematic analysis of the trials that are designed to be decentralized, describing which elements they use, where these trials are occurring, and what therapeutic areas and types of interventions they involve. While relatively few trials explicitly identify as decentralized, uptake since 2021 suggests increasing alignment with evolving regulatory terminology. The geographic concentration in high-income countries suggests broad global implementation, while variable adoption of specific decentralized components indicates areas requiring further regulatory clarity or practical guidance may be needed. Even among trials that self-identify as decentralised, terminology and reporting are inconsistent, making identification, interpretation, and synthesis of DCT evidence challenging. These findings highlight the importance of consistent terminology and reporting to support the interpretation and synthesis of decentralized clinical trials.

## Supplementary Information

Supplementary Material 1.

Supplementary Material 2.

Supplementary Material 3.

## Data Availability

The datasets used and/or analyzed during the current study are available from the corresponding author on reasonable request.
