# Supplementary material for: Adoption of ‘decentralized’ clinical trial terminology: a systematic review of self-identified decentralized clinical trials
Source: Trials. 2026 Jul 31;27:630. doi: 10.1186/s13063-026-09922-x (PMC13711692; doi:10.1186/s13063-026-09922-x)
Supplement: Supplementary file 1 [file 13063_2026_9922_MOESM3_ESM.pdf]

NIHR | National Institute for Health and Care Research

PROSPERO
International prospective register of systematic reviews

A systematic review to determine how decentralised clinical trials are being designed and conducted worldwide

Annie Wright, Joanna Gambell, Shwetha Rajaram

Citation 1 change
Annie Wright, Joanna Gambell, Shwetha Rajaram. A systematic review to determine how decentralised clinical trials are being designed and conducted worldwide. PROSPERO 2024 Available from https://www.crd.york.ac.uk/PROSPERO/view/CRD42024571898

REVIEW TITLE AND BASIC DETAILS

Review title
A systematic review to determine how decentralised clinical trials are being designed and conducted worldwide

Review objectives
To determine how decentralised clinical trials are being designed and conducted worldwide including collection and analysis methods for longitudinal data.
The specific objectives of the review are to characterise DCTs:
1. Characteristics of trials that employed decentralised approaches/methods.
2. What aspects of the trials were decentralised and how?
3. Determine if outcome data is being collected by decentralised mechanism (e.g. ePROMS or wearable devices) and methods of analysis if this is longitudinal.

Keywords
Decentralised trials

SEARCHING AND SCREENING

Searches
The search strategy has been developed in collaboration with supervisors and on previously identified papers on decentralised trials. It will include search terms that will a) identify trials as being decentralised and b) terms to search for types of study design.
The list of search terms within each concept will initially be searched using the Boolean operator ‘OR’ and then concepts will be combined using the Boolean operator ‘AND’ producing

the final search result.

The following databases will be searched for published literature:

- MEDLINE
- Embase
- CENTRAL

I will also search within two clinical trial databases listed below. I will cross-check the papers with clinical trial registries and report on the registry papers which do not yet have a protocol or final results publication. This will provide an overview of the trials in the pipeline that are self-identifying as decentralised.

- ICTRP
- ClinicalTrials.gov.

The number of articles obtained in the search period will be documented.

All papers must be in English, human studies and there will be no start date restrictions.

Study design

We will only include reports of decentralised randomised controlled trials.

For the purpose of this review, a DCT is defined as 'a clinical trial where some or all of the trial-related activities occur at locations other than traditional clinical trial sites' (1).

Inclusion criteria:

Articles that:

- Randomised clinical trials that are ongoing (where the protocol is published) or completed.
- Identify the trial as being decentralised (hybrid or fully) as per the above definition.
- Any population/country/intervention
- The trial is investigating a clinical outcome

Exclusion criteria:

Articles that:

- Are not randomised trials i.e. cohort studies.
- Are investigating a decentralised procedure i.e. the intervention being given at home is being investigated rather than IMP delivery being a decentralised method.
- Pilot/feasibility/early phase trials

1. FDA. Decentralized Clinical Trials for Drugs, Biological Products, and Devices [Internet]. FDA; 2023 [cited 2024 Mar 20]. Available from: https://www.fda.gov/regulatory-information/search-fda-guidance-documents/decentralized-clinical-trials-drugs-biological-products-and-devices

ELIGIBILITY CRITERIA

Condition or domain being studied

There is no specific disease/condition that will be studied in this review. Instead, it focuses on the specific elements used in decentralised trials and will provide a summary of the diseases/conditions that are using a decentralised trial design. This allows the reader of the review to understand what decentralisation of trial conduct is taking place across disease domains and how it can be implemented in future trials.

Population

Participants who have been enrolled in a decentralised randomised controlled trial (hybrid or fully decentralised will be included).

We will not restrict on age or therapeutic areas.

Intervention(s) or exposure(s)

This trials included will be testing any intervention that is measured with a clinical outcome and will include all randomised trials with any type of decentralised element. This might be e.g. e-consent, remote monitoring, using wearable devices to collect data.
There will be no restriction on the type of intervention that the participant receives within the trial as long as a clinical outcome is collected.

Comparator(s) or control(s)

We are interested in any intervention with a clinical outcome and therefore any comparator/control will be acceptable e.g. standard of care, sham or placebo. We will not accept single-arm studies.

OUTCOMES TO BE ANALYSED

Main outcomes

To collect information on the decentralised element(s) used in trials. Decentralised elements refer to the trial processes which have been decentralised, e.g. using e-consent rather than consenting in person. By evaluating these, we aim to determine their effectiveness in improving patient health and efficiency of clinical trials.
Elements:
• Routinely collected data
• E-consent
• Home delivery of intervention
• Physiological samples taken at home
• AE reporting online
• ePROMS
Clinicians and practitioners can use the findings of this review to implement decentralised elements that have been shown to improve patient outcomes and engagement. Commonly used elements can be integrated into the patient care pathway outside of clinical trials e.g. home sampling might not be routinely done in some disease areas but was implemented commonly in trials in the same therapeutic area and therefore could be conducted in practice. Less commonly used elements can also inform clinical practice by showing care pathways that need to be improved. By providing evidence on the effectiveness and practicality of decentralised trial elements, this review aims to inform clinical practices that enhance patient health outcomes and streamline the clinical trial process. This will improve patient experiences, increasing the likelihood of them enrolling into clinical trials and providing evidence for future treatments.

Measures of effect
Summary statistics will be presented e.g. Frequencies and proportion.

Additional outcomes

• Country of the study: Identifies regional healthcare practices that may impact the effectiveness of decentralised trial elements.
• Therapeutic area: Determines which medical conditions benefit most from decentralised trials, guiding treatment approaches.

• Study design: Evaluates whether decentralised elements are effective in both simple and complex clinical trial designs, informing trial methodologies.
• Demographics: Ensures inclusivity and generalisability of trial results across different patient populations.
• Number of screened/consented/randomised participants: Measures participant engagement and trial feasibility, crucial for successful clinical outcomes.
• Intervention and comparator details: Assesses the specific treatments and controls used, aiding in understanding their impact on patient health and which interventions can be implemented with a decentralised trial.
• If longitudinal data is collected and by what means (HSD/wearables/ePROMS): Tracks patient outcomes over time, providing insights into long-term health effects of interventions.
• Analysis methods of longitudinal data: Determines the robustness of data interpretation, ensuring accurate conclusions about patient health impacts.
• Justification for doing a DCT: Highlights the rationale for using decentralised trials, showcasing their potential benefits in improving patient care and outcomes.

DATA COLLECTION PROCESS

Data extraction (selection and coding)

After the keyword search is completed, articles will be imported into COVIDENCE, and duplicates will be removed.

Title and Abstract Screening:
• Primary Reviewer: Reviewer will screen titles and abstracts within COVIDENCE.
• Secondary Reviewer: A second reviewer will be given access to COVIDENCE and screen the same titles and abstracts, resulting in duplicate screening.
• Discrepancy Resolution: Any disagreements between the reviewers will be resolved through discussion. If consensus cannot be reached, a third reviewer will pass judgment.

Full-Text Screening:
Titles and abstracts that passed the title and abstract screening will move to the full-text screening section in COVIDENCE and full texts will be obtained and screened.
• Screening will be conducted in duplicate by both primary and secondary reviewers.
• Discrepancies will be resolved through discussion or by a third reviewer if necessary.

Data Extraction:
Data will be extracted using a specifically designed and piloted data extraction form. The extraction process includes:
• First Reviewer: extract all data from the selected studies.
• Second Reviewer: extract key data fields independently.
• The extracted data will be checked for agreement between the two reviewers.

Main Data Fields to be Extracted:
• Study Identification: Title, authors, publication year, funder
• General Study Information: Country of Study, Disease area, Indication, Primary Outcome
• Study Design: Type of study design, Number of Arms, Phase
• Population Characteristics: Age, Number of Participants
• Interventions and Comparators: Details of the interventions and control conditions.
• Decentralised Elements Used: Specific decentralised processes used
• Decentralised Data Collection: Methods for collecting longitudinal data

• Analysis Methods: Framework, Use of Repeated Measures, Type of models.
• Justification for DCT: Rationale DCT

Number of Researchers and Discrepancy Resolution

• Researchers Involved: At least two reviewers will be involved in the screening and extraction process.
• Discrepancy Resolution: Discrepancies between reviewers will be discussed and resolved. If consensus is not reached, a third reviewer will make the final decision.

Risk of bias (quality) assessment

The risk of bias for the primary outcomes of all trials included in the focused analysis will be assessed using the Cochrane risk of bias tool for randomised trials (RoB2). This will be used to provide context of the quality of the trials included.

PLANNED DATA SYNTHESIS

Strategy for data synthesis

In this review, a comprehensive data synthesis will be conducted using a combination of narrative synthesis and descriptive statistics to provide a thorough understanding of the impact of decentralised elements in clinical trials.

Descriptive Statistics:

• Summary Tables: Characteristics of the included studies, such as study design, demographics, interventions, and outcomes, will be summarised in tables.
• Software: Descriptive analyses will be conducted using R.

Data Visualisation:

• Heat Map: The use of decentralised elements across studies will be visualised using heat maps to show the frequency and combinations of elements.
• Graphical Representation: Types of data collection and longitudinal data analysis methods will be graphically represented to provide an overview.

Summary tables and heat maps will be used to interpret the frequency and distribution of decentralised elements across studies. This can also be broken down, if numbers allow, into subgroups to observe trends in therapeutic area intervention type or before and after the COVID-19 pandemic.

Narrative Synthesis:

• Presentation: A narrative synthesis will summarise the types of decentralised elements used, combinations of these elements, and types of data collection methods.
• Thematic Analysis: If data availability allows the justifications for using decentralised trial elements will be analysed thematically to identify common themes and insights. Using NVivo to complete this will be explored.

The findings from the descriptive statistics and narrative synthesis will be integrated to provide a comprehensive understanding of the impact of decentralised elements. This integration will highlight how these elements can be applied in clinical practice to improve patient health outcomes. This structured approach to data synthesis ensures that the review will provide actionable insights for clinicians and practitioners, ultimately aiming to improve patient health outcomes through the effective use of decentralised trial elements.

Analysis of subgroups or subsets

Where possible a subgroup analysis will be completed on
• Therapeutic Area: e.g. cardiovascular disease vs neurological diseases
• Intervention type: e.g. pharmacological vs behavioural
• Comparing publications before and after the COVID-19 pandemic.
Subgroup analyses will be presented in a table format.

REVIEW AFFILIATION, FUNDING AND PEER REVIEW

Review team members 1 change

- Miss Annie Wright, Imperial College London
- Joanna Gambell, The George Institute for Global Health
- Ms Shwetha Rajaram, The George Institute for Global Health

Review affiliation
Imperial College London

Funding source
NIHR Imperial Biomedical Research Centre (BRC)

Named contact
Annie Wright. Imperial Clinical Trials Unit, School of Public Health Imperial College London
White City Campus, Stadium House, 68 Wood Lane, London, W12 7RH
annie.wright@imperial.ac.uk

TIMELINE OF THE REVIEW

Review timeline
Start date: 03 June 2024. End date: 29 November 2024

Date of first submission to PROSPERO
23 July 2024

Date of registration in PROSPERO
24 July 2024

CURRENT REVIEW STAGE

Publication of review results
The intention is to publish the review once completed.The review will be published in English

Stage of the review at this submission 1 change

Review stage	Started	Completed
Pilot work	✓	✓
Formal searching/study identification	✓	✓
Screening search results against inclusion criteria	✓	✓
Data extraction or receipt of IP	✓	

Review stage	Started	Completed
Risk of bias/quality assessment	✓	✓
Data synthesis	✓	

Review status
The review is currently planned or ongoing.

ADDITIONAL INFORMATION

Additional information
Although there have been some reviews on the topic of decentralised trials there is no comprehensive overview of what is happening worldwide currently. This is important to give an overview of where decentralised trials are taking place and ensuring there is access to the type of infrastructure to carry out this type of trial in all settings. Similar reviews registered were conducted prior to the COVID-19 pandemic and therefore I believe there needs to be an update on the topic as the pandemic is likely to have directly impacted this topic. This review will show which decentralised elements are being utilised up to and after the Covid-19 pandemic which may be pivotal in them being utilised more frequently.

Collaborators

- **Professor Victoria Cornelius**, Imperial College London
- **Professor Otavio Berwanger**, The George Institute for Global Health

PROSPERO version history

- Version 1.1 published on 02 Dec 2024
- Version 1.0 published on 24 Jul 2024

Review conflict of interest
None known

Country
England

Medical Subject Headings
Humans; Wearable Electronic Devices

Revision note 1 change
Update to those involved in the review and what stage is at

Disclaimer
The content of this record displays the information provided by the review team. PROSPERO does not peer review registration records or endorse their content.

PROSPERO accepts and posts the information provided in good faith; responsibility for record content rests with the review team. The owner of this record has affirmed that the information provided is truthful and that they understand that deliberate provision of inaccurate information may be construed as scientific misconduct.

PROSPERO does not accept any liability for the content provided in this record or for its use. Readers use the information provided in this record at their own risk.

Any enquiries about the record should be referred to the named review contact
