# Supplementary material for: Adoption of ‘decentralized’ clinical trial terminology: a systematic review of self-identified decentralized clinical trials
Source: Trials. 2026 Jul 31;27:630. doi: 10.1186/s13063-026-09922-x (PMC13711692; doi:10.1186/s13063-026-09922-x)
Supplement: Supplementary file 3 [file 13063_2026_9922_MOESM2_ESM.docx]

**Supplementary Material**

**eAppendix 1 Literature Search Syntax – Medline**

**1** ((randomized controlled trial or controlled clinical trial).pt. or random*ti,ab,kf. Or placebo.ab. or drug therapy.fs. or randomly.ab. or trial.ab. or groups.ab.) not (exp animals/ not humans.sh) (Results 5,612,106)

**2** decentrali*.mp. (Results 12,386)

**3** 1 and 2 (Results 1,909)

**eAppendix 2 Literature Search Syntax – Embase**

**1** ((randomized controlled trial/ or controlled clinical trial/ or random*ti,ab,kw. or placebo.ab. or drug therapy/ or randomly.ab. or trial.ab. or groups.ab.) not (exp animals/ not humans/) (Results 6,066,845)

**2** decentrali*.mp. (Results 15,264)

**3** 1 and 2 (Results 2,053)

**eAppendix 3 Literature Search Syntax – Central**

**1** decentrali* (Results 466)

**2** randomized controlled trial:pt (Results 284,723)

**3** controlled clinical trial:pt (Results 354,121)

**4** random*:ti,ab (Results 1,289,345)

**5** placebo:ab (Results 362,518)

**6** randomly:ab (Results 336,514)

**7** trial:ab (Results 635,107)

**8** groups:ab (Results 633,007)

**9** 2 OR 3 OR 4 OR 5 OR 6 OR 7 OR 8 (Results 1,677,884)

**10** 1 AND 9 (Results 430)

**eTable 1 Data extraction categories and fields**

**General**

Reviewer Name

Date of Extraction

Name of Trial

Study Number

Year of publication

Link to the paper

Journal

Primary Author Surname

**Set up and design**

Funder

Country(ies) study run in

Primary country

Other Countries

Trial Registration Link

First posted on a clinical trial registration

Status

Any linked papers

Recruitment

Trial Dates

Start Date (recruitment start date) – if the paper is a protocol these will be left empty

End Date (recruitment end date, primary completion) – if the paper is a protocol these will be left empty

Disease area

Indication

**Study Methods**

Design Features

Parallel arm

Adaptive Trial Design

If yes, adaptive design description

if a platform/GSD was used was it a frequentist or Bayesian framework

Type of Randomisation

Arms

Phase

Primary outcome

Description

Was the study **designed** on one endpoint or taking advantage of longitudinal measurements

**Population Characteristics**

Age

Inclusion

Actual (only in main papers)

Sample Size

Estimated/planned

Recruited/Assessed for eligibility/screened

Randomised

**Interventions**

What is the intervention

Type of intervention

What is the mode of delivery of the intervention?

Duration of the intervention

What is the comparator

**Decentralised elements**

Thinking about screening: did the trial use routine data sources to identify participants

Type of routine data

Did the trial use e-consent?

Was the intervention delivered outside its typical context?

If yes above, where?

How did follow up study visits take place

Were physiological samples collected

If yes were they collected at home

If yes what type of physiological samples were collected at home

Were biological samples collected

If yes were they collected at home

If yes what type of biological samples were collected at home

Was the trial centrally monitored e.g. remote monitoring visits by the **trial sponsor**

Was the IMP (or device) delivered to the participants home?

Did the trial use an online AE Reporting system

Other DCT element not mentioned above

**Decentralised Data Collection**

How was the primary outcome data collected

Were PROMS collected at all

Method of PROM collection

If PROM was collected for the primary outcome what was the type of PROM

Was routinely collected data used at all

What routine data source(s) were used

If used for the primary outcome what variables were extracted

Was any data collected by a device or sensor

If yes was it for the primary outcome

If yes what was the type of device

**Analysis**

Bayesian or Frequentist framework for primary outcome

If Bayesian – did they use informative or vague priors

Were repeated measures collected for their primary outcome (where relevent)

Did they make use of longitudinal data over time in the analysis

How were the repeated measures analysed

What type of model was used

Was a random intercept included

Was an interaction term included

Type of interaction term

Type of covariance matrix structure

**Other**

Justification of doing a DCT

Comments

**eTable 2 Resources used for data extraction**

| **Akechi 2024** | UMIN000054583 |
| --- | --- |
|  | Akechi T, Furukawa TA, Hashimoto H, Harada Y, Ito Y, Furukawa Y, et al. Smartphone-based distress screening, information provision, and psychotherapy for reducing psychological distress among AYA cancer survivors: protocol for a fully decentralized multicenter randomized controlled clinical trial. Jpn J Clin Oncol. 2024 Aug 28;hyae111 |
| **Akechi 2024** | UMIN000041536 |
|  | Akechi T, Furukawa TA, Noma H, Iwata H, Toyama T, Higaki K, et al. Optimizing smartphone psychotherapy for depressive symptoms in patients with cancer: Multiphase optimization strategy using a decentralized multicenter randomized clinical trial (J-SUPPORT 2001 Study). Psychiatry Clin Neurosci. 2024;78(6):353–61. |
|  | Uchida M, Furukawa TA, Yamaguchi T, Imai F, Momino K, Katsuki F, et al. Optimization of smartphone psychotherapy for depression and anxiety among patients with cancer using the multiphase optimization strategy (MOST) framework and decentralized clinical trial system (SMartphone Intervention to LEssen depression/Anxiety and GAIN resilience: SMILE AGAIN project): a protocol for a randomized controlled trial. Trials. 2023 May 22;24(1):344 |
|  | SAP available as supplementary to the main paper |
| **Akechi 2022** | UMIN000031140 |
|  | Akechi T, Yamaguchi T, Uchida M, Imai F, Momino K, Katsuki F, et al. Smartphone Psychotherapy Reduces Fear of Cancer Recurrence Among Breast Cancer Survivors: A Fully Decentralized Randomized Controlled Clinical Trial (J-SUPPORT 1703 Study). J Clin Oncol Off J Am Soc Clin Oncol. 2022;41(5):1069–78 |
|  | Protocol and SAP available as supplementary to the main paper |
| **Best 2023** | NCT04586348 |
|  | Best KP, Gould JF, Makrides M, Sullivan T, Cheong J, Zhou SJ, et al. Prenatal iodine supplementation and early childhood neurodevelopment: the PoppiE trial – study protocol for a multicentre randomised controlled trial. BMJ Open. 2023 May 1;13(5):e071359 |
| **Boucher 2024** | NCT04567888 |
|  | Boucher EM, Ward H, Miles CJ, Henry RD, Stoeckl SE. Effects of a Digital Mental Health Intervention on Perceived Stress and Rumination in Adolescents Aged 13 to 17 Years: Randomized Controlled Trial. J Med Internet Res. 2024 Mar 29;26:e54282 |
|  | Boucher EM, Ward HE, Stafford JL, Parks AC. Effects of a Digital Mental Health Program on Perceived Stress in Adolescents Aged 13-17 Years: Protocol for a Randomized Controlled Trial. JMIR Res Protoc 2021;10(4):e25545 |
| **Bramante 2023** | NCT04510194 |
|  | Bramante CT, Buse JB, Liebovitz DM, Nicklas JM, Puskarich MA, Cohen K, et al. Outpatient treatment of COVID-19 and incidence of post-COVID-19 condition over 10 months (COVID-OUT): a multicentre, randomised, quadruple-blind, parallel-group, phase 3 trial. Lancet Infect Dis. 2023 Oct;23(10):1119–29. |
|  | Protocol available as supplementary to the main paper |
| **Chung 2024** |  |
|  | Chung MK, Kim DH, Park JI, Lee S, Park HC, Kim K, et al. Adaptive Nutrition Intervention Stabilizes Serum Phosphorus Levels in Hemodialysis Patients: A Multicenter Decentralized Clinical Trial Using Real-World Data. J Ren Nutr. 2024 Jan 1;34(1):47–57 |
| **Gibson 2023** | NCT04276441 |
|  | Gibson CM, Steinhubl S, Lakkireddy D, Turakhia MP, Passman R, Jones WS, et al. Does early detection of atrial fibrillation reduce the risk of thromboembolic events? Rationale and design of the Heartline study. Am Heart J. 2023 May;259:30–41 |
| **Huckvale 2023** | ACTRN12621001223820 |
|  | Huckvale K, Hoon L, Stech E, Newby JM, Zheng WY, Han J, et al. Protocol for a bandit-based response adaptive trial to evaluate the effectiveness of brief self-guided digital interventions for reducing psychological distress in university students: the Vibe Up study. BMJ Open. 2023 Apr 1;13(4):e066249 |
| **Kaizer 2023** | NCT04372628 |
|  | Kaizer AM, Shapiro NI, Wild J, Brown SM, Cwik BJ, Hart KW, et al. Lopinavir/ritonavir for treatment of non-hospitalized patients with COVID-19: a randomized clinical trial. Int J Infect Dis. 2023 Mar 1;128:223–9 |
|  | Kaizer AM, Wild J, Lindsell CJ, Rice TW, Self WH, Brown S, et al. Trial of Early Antiviral Therapies during Non-hospitalized Outpatient Window (TREAT NOW) for COVID-19: a summary of the protocol and analysis plan for a decentralized randomized controlled trial. Trials. 2022 Apr 8;23(1):273 |
| **Kolobaric 2024** | NCT05511818 |
|  | Kolobaric A, Saleska J, Hewlings SJ, Bryant C, Colwell CS, D’Adamo CR, et al. A Randomized, Double-Blind, Placebo-Controlled Trial to Assess the Effectiveness and Safety of Melatonin and Three Formulations of Floraworks Proprietary TruCBN^TM^ for Improving Sleep. Pharmaceuticals. 2024 Aug;17(8):977. |
| **Kolobaric 2023** | NCT05511818 |
|  | Kolobaric A, Hewlings SJ, Bryant C, Colwell CS, R. D’Adamo C, Rosner B, et al. A Randomized, Double-Blind, Placebo-Controlled Decentralized Trial to Assess Sleep, Health Outcomes, and Overall Well-Being in Healthy Adults Reporting Disturbed Sleep, Taking a Melatonin-Free Supplement. Nutrients. 2023 Jan;15(17):3788 |
| **Krumholz 2024** | NCT05668091 |
|  | Krumholz HM, Sawano M, Bhattacharjee B, Caraballo C, Khera R, Li SX, et al. The PAX LC Trial: A Decentralized, Phase 2, Randomized, Double-Blind Study of Nirmatrelvir/Ritonavir Compared with Placebo/Ritonavir for Long COVID. Am J Med. 2024 May;S0002934324002717 |
| **LaForce 2024** | NCT05505734 |
|  | LaForce C, Albers FC, Cooper M, Danilewicz A, Dunsire L, Rees R, et al. A Fully Decentralized Randomized Controlled Study of As-Needed Albuterol-Budesonide Fixed-Dose Inhaler in Mild Asthma: The BATURA Study Design. J Asthma Allergy. 2024 Aug 23;17:801–11. |
| **Lee 2021** | NCT03870997 |
|  | Lee JL, Foschini L, Kumar S, Juusola J, Liska J, Mercer M, et al. Digital intervention increases influenza vaccination rates for people with diabetes in a decentralized randomized trial. Npj Digit Med. 2021 Sep 17;4(1):1–8. |
| **Lu 2023** | ChiCTR2200059218 |
|  | Lu Y, Li Y, Huang Y, Zhang X, Wang J, Wu L, et al. Effects and Mechanisms of a Web- and Mobile-Based Acceptance and Commitment Therapy Intervention for Anxiety and Depression Symptoms in Nurses: Fully Decentralized Randomized Controlled Trial. J Med Internet Res. 2023 Nov 27;25(1):e51549 |
| **Mackenzie 2022** | EudraCT (2011-001968-21) and ISRCTN (18157641) |
|  | Mackenzie IS, Rogers A, Poulter NR, Williams B, Brown MJ, Webb DJ, et al. Cardiovascular outcomes in adults with hypertension with evening versus morning dosing of usual antihypertensives in the UK (TIME study): a prospective, randomised, open-label, blinded-endpoint clinical trial. The Lancet. 2022 Oct 22;400(10361):1417–25 |
|  | Protocol and SAP available in the supplementary appendix |
| **Marshall 2022** | NCT04584645 |
|  | Marshall NJ, Lee JL, Schroeder J, Lee WN, See J, Madjid M, et al. Influence of Digital Intervention Messaging on Influenza Vaccination Rates Among Adults With Cardiovascular Disease in the United States: Decentralized Randomized Controlled Trial. J Med Internet Res. 2022 Oct 7;24(10):e38710. |
| **Naggie 2022** | NCT04885530 |
|  | Naggie S, Boulware DR, Lindsell CJ, Stewart TG, Gentile N, Collins S, et al. Effect of Ivermectin vs Placebo on Time to Sustained Recovery in Outpatients With Mild to Moderate COVID-19: A Randomized Clinical Trial. JAMA. 2022 Oct 25;328(16):1595–603. |
| **Naik 2024** | NCT05430152 |
|  | Naik H, Cooke E, Boulter T, Dyer R, Bone JN, Tsai M, et al. Low-dose naltrexone for post-COVID fatigue syndrome: a study protocol for a double-blind, randomised trial in British Columbia. BMJ Open. 2024 May;14(5):e085272. |
| **Schroder 2022** | DRKS00024657 |
|  | Schröder T, Brudermann HCB, Kühn G, Sina C, Thaçi D, Nitschke M, et al. Efficacy of the Digital Therapeutic sinCephalea in the prophylaxis of migraine in patients with episodic migraine: study protocol for a digital, randomized, open-label, standard treatment controlled trial. Trials. 2022 Dec 12;23(1):997. |
| **Shortt 2024** | ACTRN12621001096842 |
|  | Shortt G, Shortt N, Bird G, Kerse K, Lieffering N, Martin A, et al. Mānuka oil based ECMT-154 versus vehicle control for the topical treatment of eczema: study protocol for a randomised controlled trial in community pharmacies in Aotearoa New Zealand. BMC Complement Med Ther. 2024 Jan 29;24(1):61. |
| **Shortt 2022** | ACTRN12618001754235 |
|  | Shortt N, Martin A, Kerse K, Shortt G, Vakalalabure I, Barker L, et al. Efficacy of a 3% Kānuka oil cream for the treatment of moderate-to-severe eczema: A single blind randomised vehicle-controlled trial. eClinicalMedicine. 2022 Sep 1;51:101561 |
|  | Protocol available as supplementary to the main paper |
| **Silberman 2022** | NCT04609644 |
|  | Silberman J, Sarlati S, Harris B, Bokhari W, Boushey H, Chesnutt A, et al. A digital approach to asthma self-management in adults: Protocol for a pragmatic randomized controlled trial. Contemp Clin Trials. 2022 Nov;122:106902 |
| **Silverberg 2024** | NCT04091087 |
|  | Silverberg JI, Kirsner RS, Margolis DJ, Tharp M, Myers DE, Annis K, et al. Efficacy and safety of crisaborole ointment, 2%, in participants aged ≥45 years with stasis dermatitis: Results from a fully decentralized, randomized, proof-of-concept phase 2a study. J Am Acad Dermatol. 2024 May;90(5):945–52 |
| **Spertus 2021** | NCT04252287 |
|  | Spertus JA, Birmingham MC, Butler J, Lingvay I, Lanfear DE, Abbate A, et al. Novel Trial Design: CHIEF-HF. Circ Heart Fail. 2021 Mar;14(3):e007767 |
| **Yao 2024** | NCT05923359 |
|  | Yao X, Attia ZI, Behnken EM, Hart MS, Inselman SA, Weber KC, et al. Realtime Diagnosis from Electrocardiogram Artificial Intelligence-Guided Screening for Atrial Fibrillation with Long Follow-Up (REGAL): Rationale and design of a pragmatic, decentralized, randomized controlled trial. Am Heart J. 2024;267(0370465):62–9 |
| **Yokoi 2024** | jRCTs032230339 |
|  | Yokoi Y., Inagawa T., Yamada Y., Matsui M., Tomizawa A., Noda T. A randomized sham-controlled trial of transcranial and intranasal photobiomodulation in Japanese patients with mild cognitive impairment and mild dementia due to Alzhimer’s disease: a protocol. medRxiv [Internet]. 2023;((Yokoi, Inagawa, Yamada, Matsui, Tomizawa, Noda) National Center of Neurology and Psychiatry, Japan). Available from: https://www.medrxiv.org/ |

**eTable 3 Information on included trials**

|  | **Protocol**  **(N=13)** | **Main Paper**  **(N=15)** | **Overall**  **(N=28)** |
| --- | --- | --- | --- |
| **Year of Publication** |  |  |  |
| 2021 | 1 (8%) | 1 (7%) | 2 (7%) |
| 2022 | 2 (15%) | 5 (33%) | 7 (25%) |
| 2023 | 3 (23%) | 4 (27%) | 7 (25%) |
| 2024 | 7 (54%) | 5 (33%) | 12 (43%) |
| **Year of registration on a clinical trial registry** |  |  |  |
| 2014 | 0 (0%) | 1 (7%) | 1 (4%) |
| 2015 | 0 (0%) | 0 (0%) | 0 (0%) |
| 2016 | 0 (0%) | 0 (0%) | 0 (0%) |
| 2017 | 0 (0%) | 0 (0%) | 0 (0%) |
| 2018 | 0 (0%) | 2 (13%) | 2 (7%) |
| 2019 | 0 (0%) | 2 (13%) | 2 (7%) |
| 2020 | 4 (31%) | 5 (33%) | 9 (32%) |
| 2021 | 3 (23%) | 1 (7%) | 4 (14%) |
| 2022 | 3 (23%) | 4 (27%) | 7 (25%) |
| 2023 | 2 (15%) | 0 (0%) | 2 (7%) |
| 2024 | 1 (8%) | 0 (0%) | 1 (4%) |
| **Country Study Conducted In** |  |  |  |
| Australia | 2 (15%) | 0 (0%) | 2 (7%) |
| Canada | 1 (8%) | 0 (0%) | 1 (4%) |
| China | 0 (0%) | 1 (7%) | 1 (4%) |
| Germany | 1 (8%) | 0 (0%) | 1 (4%) |
| Japan | 2 (15%) | 2 (13%) | 4 (14%) |
| New Zealand | 1 (8%) | 1 (7%) | 2 (7%) |
| South Korea | 0 (0%) | 1 (7%) | 1 (4%) |
| United Kingdom | 0 (0%) | 1 (7%) | 1 (4%) |
| United States | 6 (46%) | 9 (60%) | 15 (54%) |
| **Disease Area** |  |  |  |
| Cardiovascular | 3 (23%) | 1 (7%) | 4 (14%) |
| Endocrine | 0 (0%) | 1 (7%) | 1 (4%) |
| Infectious Diseases | 1 (8%) | 3 (20%) | 4 (14%) |
| Mental Health | 2 (15%) | 4 (27%) | 6 (21%) |
| Neurology | 4 (31%) | 0 (0%) | 4 (14%) |
| Respiratory | 2 (15%) | 2 (13%) | 4 (14%) |
| Other | 1 (8%) | 4 (27%) | 5 (18%) |
| **Type of Intervention** |  |  |  |
| Behavioural | 2 (15%) | 4 (27%) | 6 (21%) |
| Combined | 1 (8%) | 1 (7%) | 2 (7%) |
| Educational | 0 (0%) | 3 (20%) | 3 (11%) |
| Nutritional | 1 (8%) | 0 (0%) | 1 (4%) |
| Pharmacological | 5 (38%) | 7 (47%) | 12 (43%) |
| Technological | 4 (31%) | 0 (0%) | 4 (14%) |
| **Type of Primary Outcome** |  |  |  |
| Clinical Event | 2 (15%) | 4 (27%) | 6 (21%) |
| Composite | 1 (8%) | 1 (7%) | 2 (7%) |
| Physio/Biological | 0 (0%) | 1 (7%) | 1 (4%) |
| Quality of Life | 7 (54%) | 6 (40%) | 13 (46%) |
| Symptom Based | 2 (15%) | 3 (20%) | 5 (18%) |
| Time to Event | 1 (8%) | 0 (0%) | 1 (4%) |
| **Study Design** |  |  |  |
| Parallel Arm | 12 (92%) | 13 (87%) | 25 (89%) |
| Platform | 1 (8%) | 2 (13%) | 3 (11%) |
| **Type of Follow-Up** |  |  |  |
| Fully Remote | 9 (69%) | 11 (73%) | 20 (71%) |
| Hybrid | 4 (31%) | 3 (20%) | 7 (25%) |
| Unclear | 0 (0%) | 1 (7%) | 1 (4%) |
| **Approximate Trial Recruitment Period (months)** |  |  |  |
| Median [Min, Max] | - | 11.1 [0.7, 77.6] | 11.1 [0.7, 77.6] |
| Q1-Q3 | - | 5.86-22.3 | 5.9-22.3 |
| Missing | - | 3 (20%) | 16 (57%) |
| **Estimated Sample Size** |  |  |  |
| Median [Min, Max] | 754 [30.0, 28000] | 900 [66.0, 49000] | 900 [30.0, 49000] |
| Q1-Q3 | 160-1440 | 375-1460 | 192-1470 |
| Missing | 0 (0%) | 1 (6.7%) | 1 (3.6%) |
| **Screened Sample Size** |  |  |  |
| Median [Min, Max] | - | 1020 [125, 49100] | 1020 [125, 49100] |
| Q1-Q3 | - | 457-12100 | 457-12100 |
| Missing | - | 0 (0%) | 13 (46.4%) |
| **Randomised Sample Size** |  |  |  |
| Median [Min, Max] | - | 448 [66.0, 49100] | 448 [66.0, 49100] |
| Q1-Q3 | - | 264-1620 | 264-1620 |
| Missing | - | 0 (0%) | 13 (46.4%) |
| **Recruitment Status** |  |  |  |
| Equal | - | 2 (13%) | 2 (7%) |
| Overrecruited | - | 8 (53%) | 8 (29%) |
| Underrecruited | - | 4 (27%) | 4 (14%) |
| Missing | - | 1 (6.7%) | 14 (50.0%) |
| **Percentage over recruitment** |  |  |  |
| Median [Min, Max] | - | 106 [100, 388] | 106 [100, 388] |
| Q1-Q3 | - | 102-117 | 102-117 |
| Not applicable | - | 7 (46.7%) | 20 (71.4%) |
| **Percentage under recruitment** |  |  |  |
| Median [Min, Max] | - | 58.9 [44.1, 68.9] | 58.9 [44.1, 68.9] |
| Q1-Q3 | - | 48.4-68.2 | 48.4-68.2 |
| Not applicable | - | 11 (73.3%) | 24 (85.7%) |

**eTable 4 DCT elements used in the trials**

|  | **Main Paper**  **(N=15)** | **Protocol**  **(N=13)** | **Overall**  **(N=28)** |
| --- | --- | --- | --- |
| **Were Electronic Health Records used for Screening?** |  |  |  |
| Not Reported/Unclear | 14 (93%) | 7 (54%) | 21 (75%) |
| Yes | 1 (7%) | 6 (46%) | 7 (25%) |
| **Was consent collected?** |  |  |  |
| No | 2 (13%) | 0 (0%) | 2 (7%) |
| Yes | 13 (87%) | 13 (100%) | 26 (93%) |
| **If yes, was E-Consent used?** |  |  |  |
| No | 1 (8%) | 1 (8%) | 2 (8%) |
| Not Reported/Unclear | 2 (15%) | 2 (15%) | 4 (15%) |
| Yes | 10 (77%) | 10 (77%) | 20 (77%) |
| **Were physiological samples collected?** |  |  |  |
| No | 10 (67%) | 6 (46%) | 16 (57%) |
| Yes | 5 (33%) | 7 (54%) | 12 (43%) |
| **If yes were physiological samples collected at the participant's home?** |  |  |  |
| Not Reported/Unclear | 1 (20%) | 0 (0%) | 1 (8%) |
| Yes | 4 (80%) | 7 (100%) | 11 (92%) |
| **Were biological samples collected?** |  |  |  |
| No | 12 (80%) | 6 (46%) | 18 (64%) |
| Yes | 3 (20%) | 7 (54%) | 10 (36%) |
| **If yes were biological samples collected at the participant's home?** |  |  |  |
| Not Reported/Unclear | 1 (33%) | 0 (0%) | 1 (10%) |
| Yes | 2 (67%) | 5 (71%) | 7 (70%) |
| No | 0 (0%) | 2 (29%) | 2 (20%) |
| **Were Electronic Health Records used for Outcome Collection?** |  |  |  |
| Not Reported/Unclear | 11 (73%) | 8 (62%) | 19 (68%) |
| Yes | 4 (27%) | 5 (38%) | 9 (32%) |
| **Was a Device used for Outcome Collection?** |  |  |  |
| Not Reported/Unclear | 5 (33%) | 2 (15%) | 7 (25%) |
| Yes | 10 (67%) | 11 (85%) | 21 (75%) |
| **Was centralised monitoring used?** |  |  |  |
| Not Reported/Unclear | 12 (80%) | 10 (77%) | 22 (79%) |
| Yes | 3 (20%) | 3 (23%) | 6 (21%) |
| **Was an IMP/Device needed to be delivered?** |  |  |  |
| No | 7 (47%) | 2 (15%) | 9 (32%) |
| Yes | 8 (53%) | 11 (85%) | 19 (68%) |
| **Was IMP Delivery direct to the participant's home?** |  |  |  |
| No | 1 (12%) | 1 (9%) | 2 (11%) |
| Yes | 7 (88%) | 8 (73%) | 15 (79%) |
| Not Reported/Unclear | 0 (0%) | 2 (18%) | 2 (11%) |
| **Were Adverse Events collected?** |  |  |  |
| No | 3 (20%) | 2 (15%) | 5 (18%) |
| Yes | 12 (80%) | 11 (85%) | 23 (82%) |
| **Was an online AE Platform used?** |  |  |  |
| No | 1 (8%) | 1 (9%) | 2 (9%) |
| Not Reported/Unclear | 7 (58%) | 4 (36%) | 11 (48%) |
| Yes | 4 (33%) | 6 (55%) | 10 (43%) |

**eTable 5 Reasons trial identified from a clinical trial registry was ineligible**

|  | **Overall**  **(N=92)** |
| --- | --- |
| **Reason for ineligibility** |  |
| Can not find a record | 1 (1%) |
| Does not mention being decentralised | 1 (1%) |
| Testing decentralisation | 37 (40%) |
| Wrong Context | 29 (32%) |
| Wrong Design | 8 (9%) |
| Wrong Outcome | 5 (5%) |
| Wrong Phase | 11 (12%) |

**eTable 6 Information on trials considered eligible from a clinical trial registry**

|  | **Overall**  **(N=28)** |
| --- | --- |
| **The reason it wasn't included in the review** |  |
| Commentary Paper | 1 (4%) |
| Did not use decentralised in publication | 1 (4%) |
| No publication | 8 (29%) |
| Ongoing | 15 (54%) |
| Only abstract appeared in the search | 1 (4%) |
| Terminated | 2 (7%) |
| **Which database the registry information was extracted from** |  |
| ClinicalTrials.gov | 26 (93%) |
| ICTRP | 2 (7%) |
| **Type of Intervention** |  |
| Behavioural | 6 (21%) |
| Combined | 6 (21%) |
| Nutritional | 2 (7%) |
| Pharmacological | 7 (25%) |
| Technological | 3 (11%) |
| Biological | 1 (4%) |
| Other | 3 (11%) |
| **Continent Study Conducted In** |  |
| North America | 13 (46%) |
| Asia | 7 (25%) |
| Europe | 5 (18%) |
| South America | 1 (4%) |
| Multiple | 1 (4%) |
| Not Reported | 1 (4%) |

**eTable 7 Risk of Bias**

|  | **Main Paper**  **(N=15)** | **Protocol**  **(N=13)** | **Overall**  **(N=28)** |
| --- | --- | --- | --- |
| **Risk of bias arising from the randomization process** |  |  |  |
| High | 0 (0%) | 0 (0%) | 0 (0%) |
| Some concerns | 0 (0%) | 0 (0%) | 0 (0%) |
| Low | 15 (100%) | 13 (100%) | 28 (100%) |
| **Risk of bias due to deviations from the intended interventions** |  |  |  |
| High | 5 (33%) | 6 (46%) | 11 (39%) |
| Some concerns | 4 (27%) | 1 (8%) | 5 (18%) |
| Low | 6 (40%) | 6 (46%) | 12 (43%) |
| **Risk of bias in the measurement of the outcome** |  |  |  |
| High | 0 (0%) | 0 (0%) | 0 (0%) |
| Some concerns | 5 (33%) | 5 (38%) | 10 (36%) |
| Low | 10 (67%) | 8 (62%) | 18 (64%) |
| **Overall** |  |  |  |
| High | 3 (20%) | 3 (23%) | 6 (21%) |
| Some concerns | 6 (40%) | 3 (23%) | 9 (32%) |
| Low | 6 (40%) | 7 (54%) | 13 (46%) |
